# From the Pain Matrix to Functional Networks: A Narrative Review of Chronic Pain Mechanisms Across Adult and Pediatric Populations with Emerging AI Perspectives

**DOI:** 10.3390/brainsci16060639

**Published:** 2026-06-15

**Authors:** Marco Cascella, Daniela Siano, Mauro D’Amora, Corrado Cecchetti, Alessandro Vittori, Maria Romano, Vittorio Santoriello

**Affiliations:** 1 Unit of Anesthesiology, Intensive Care Medicine, and Pain Medicine, Department of Medicine, Surgery, and Dentistry, University of Salerno, 84082 Salerno, Italy; 2 Department of Anesthesia, Critical Care and Pain Medicine, ARCO, Ospedale Pediatrico Bambino Gesù IRCCS, Piazza S. Onofrio 4, 00165 Rome, Italy; 3 Department of Electrical Engineering and Information Technology, University of Naples Federico II, 80138 Naples, Italy; 4 Institute of Biomedical and Neural Engineering, Reykjavik University, 102 Reykjavik, Iceland

**Keywords:** chronic pain, pediatric pain, pain chronification, neuroplasticity, functional connectivity, brain networks, artificial intelligence, machine learning, digital biomarkers, precision pain medicine

## Abstract

**Background:** While region-based models have informed pain neuroscience, chronic pain is now increasingly conceptualized as a network disorder. This narrative review aimed to critically examine the conceptual evolution of chronic pain models from region-based representations toward large-scale functional network frameworks across adult and pediatric populations while exploring how emerging artificial intelligence (AI)-driven approaches may support future precision pain medicine. **Methods:** A structured literature search was performed in PubMed, Scopus, and Web of Science, focusing on the scientific output addressing adult and pediatric chronic pain, pain-related neuroplasticity, functional network alterations, neuromodulation, and AI-based applications in pain medicine. **Results:** The reviewed literature supports a progressive conceptual shift from region-based representations of pain toward network-oriented models involving dysfunctional interactions among the salience, default mode, central executive, and sensorimotor networks. Although emerging evidence suggests developmental network alterations in pediatric chronic pain, current conclusions remain limited by the relative scarcity of longitudinal neuroimaging studies. Emerging AI applications demonstrate promising potential for objective pain assessment, trajectory prediction, and personalized therapeutic decision-making. **Conclusions:** The transition from the pain matrix to functional network models represents one of the most important conceptual advances in contemporary pain neuroscience. A network-based perspective may accelerate AI-enabled pain biomarkers and individualized interventions.

## 1. Introduction

Pain is a multidimensional, inherently subjective experience shaped by the dynamic interplay of sensory, emotional, cognitive, and contextual factors. According to the International Association for the Study of Pain (IASP), pain is “an unpleasant sensory and emotional experience associated with, or resembling that associated with, actual or potential tissue damage” [1]. Although nociception and pain are closely related, they are distinct biological and experiential phenomena, underscoring the complexity of defining the mechanisms underlying pain persistence and chronification. Importantly, pain is not a passive readout of peripheral nociceptive input but an actively constructed perceptual experience shaped by prior expectations, contextual interpretation, emotional state, and ongoing brain network dynamics. This perspective has progressively shifted pain research from stimulus-centered models toward predictive, network-based, and systems neuroscience frameworks [2,3].

In recent decades, chronic pain has emerged as a major global health challenge affecting individuals across the lifespan, from childhood to older adulthood. In both adult and pediatric populations, persistent pain is associated with substantial functional impairment, psychological distress, reduced quality of life, and increased healthcare utilization [1,2]. Importantly, pediatric chronic pain should not be regarded simply as an earlier manifestation of adult pain conditions. Instead, pain occurring during critical phases of brain maturation may interfere with neurodevelopmental trajectories, affect emotional and cognitive processing, and potentially predispose individuals to long-term alterations in pain perception and behavioral adaptation [4,5].

Traditional distinctions between acute and chronic pain have largely relied on temporal criteria. However, duration alone has proven insufficient to capture the biological complexity of pain chronification, particularly in conditions in which maladaptive pain mechanisms emerge early or persist beyond tissue healing. Growing evidence suggests that the transition from acute to chronic pain is not a passive consequence of prolonged nociceptive input, but rather an active, multidimensional process involving maladaptive neuroplasticity at peripheral, spinal, and supraspinal levels of the nervous system [6].

As emphasized by previous mechanistic studies, pain chronification reflects dynamic interactions among sensory-discriminative, affective–motivational, cognitive, and contextual domains, leading to persistent reorganization of neural circuits involved in salience detection, emotional appraisal, learning, memory, and behavioral regulation [6,7]. These processes may be particularly relevant during childhood and adolescence, when large-scale functional brain networks, including the default mode, salience, and executive control networks, are still undergoing structural and functional maturation.

Historically, neuroimaging studies have attempted to localize pain processing within discrete cortical and subcortical regions, leading to the formulation of the so-called pain matrix [8]. Although this framework has substantially contributed to our understanding of nociceptive processing, accumulating evidence has challenged its specificity and has progressively shifted attention toward network-based models of pain. Chronic pain is now increasingly conceptualized as a disorder of distributed functional networks rather than isolated brain regions, offering a more integrated explanation for symptom persistence, cognitive dysfunction, emotional comorbidities, and interindividual variability [9].

At the same time, the progressive recognition of chronic pain as a multiscale disorder involving dynamic interactions among neural, behavioral, autonomic, and contextual dimensions has generated unprecedented analytical complexity. Conventional statistical and reductionist approaches may be insufficient to fully capture these high-dimensional and temporally evolving interactions, particularly when considering developmental variability across the lifespan. In this context, advances in artificial intelligence (AI), machine learning (ML), and multimodal data integration are creating new opportunities to translate mechanistic insights into clinically actionable strategies. By combining neuroimaging, electrophysiology, wearable biosensors, digital biomarkers, and patient-reported outcomes, AI may facilitate objective pain phenotyping, prediction of clinical trajectories, and personalized therapeutic decision-making across both adult and pediatric populations [10].

Accordingly, integrating systems neuroscience, developmental neurobiology, and AI-based analytical approaches may provide a unified framework for understanding pain chronification across the lifespan and for translating mechanistic insights into clinically actionable precision pain medicine strategies [11]. Therefore, in this narrative review, we explore the evolution of pain concepts from the traditional pain matrix model toward contemporary network-based frameworks, discuss the implications of these perspectives across different developmental stages, and examine how AI-driven approaches may facilitate the translation of network-level neuroscience into objective, personalized, and clinically meaningful pain assessment strategies.

## 2. Methods

This manuscript was developed as a narrative review in accordance with the methodological principles proposed by the SANRA (Scale for the Assessment of Narrative Review Articles) framework [12]. The review was designed to explore the neurobiological mechanisms underlying chronic pain across the lifespan, including both adult and pediatric populations, with particular focus on the evolution from region-based models such as the pain matrix toward large-scale functional network perspectives, and on the emerging role of AI in pain phenotyping, assessment, and personalized management.

A structured literature search was performed in PubMed, Scopus, and Web of Science from database inception through March 2026. Search terms included combinations of the following keywords: “chronic pain”, “persistent pain”, “pediatric pain”, “children”, “adolescents”, “pain matrix”, “functional connectivity”, “default mode network”, “salience network”, “central executive network”, “pain chronification”, “neuroplasticity”, “artificial intelligence”, “machine learning”, “deep learning”, and “pain biomarkers”. Boolean operators (“AND”, “OR”) were used to optimize retrieval.

Eligible publications included original studies, clinical investigations, neuroimaging studies, meta-analyses, systematic reviews, narrative reviews, and methodological papers published in English, focusing on adult and pediatric chronic pain, pain-related neuroplasticity, functional network alterations, neuromodulation, and AI applications in pain medicine. Additional relevant studies were identified through manual screening of reference lists and expert-driven selection based on clinical and scientific relevance.

Evidence synthesis followed an iterative interpretative process consistent with narrative review methodology. Priority was given to seminal mechanistic studies, influential conceptual frameworks, and recent systematic reviews and meta-analyses that significantly contributed to the understanding of pain chronification, functional network alterations, developmental neuroscience, and AI applications in pain medicine. When available, evidence from higher-level sources was preferentially considered to support conceptual synthesis and interpretation. Following critical appraisal, the selected literature was thematically organized into four major conceptual domains: (i) conceptual foundations of pain chronification across the lifespan; (ii) strengths and limitations of region-based models, including the pain matrix; (iii) large-scale functional network alterations underlying chronic pain; and (iv) emerging AI-based applications for precision pain assessment and tailored therapeutic strategies.

## 3. Mechanisms of Pain Chronification

Accumulating evidence suggests that the transition from acute to chronic pain is an active and multidimensional neurobiological process [13]. Following tissue injury, inflammatory mediators such as nerve growth factor (NGF), tumor necrosis factor-α (TNF-α), interleukin-1β (IL-1β), prostaglandins, and chemokines initiate peripheral sensitization by directly activating nociceptors and lowering neuronal activation thresholds [14]. In particular, NGF has been shown to induce phosphorylation-dependent sensitization of transient receptor potential vanilloid-1 (TRPV1) channels while simultaneously activating mitogen-activated protein kinase (MAPK) pathways, resulting in increased expression of voltage-gated sodium channels Nav1.7 and Nav1.8, ultimately promoting sustained neuronal hyperexcitability [15]. Infiltrating macrophages further amplify peripheral nociceptive signaling through persistent release of pro-inflammatory cytokines. As nociceptive signaling persists, maladaptive neuroimmune interactions progressively involve the spinal cord and supraspinal structures. Activated microglia within the dorsal horn trigger Toll-like receptor 4 (TLR4)/MyD88-dependent signaling cascades, promoting long-term potentiation-like mechanisms in nociceptive neurons and facilitating central sensitization [16]. Additionally, this complex process of pain chronification is sustained by persistent transcriptional and epigenetic reprogramming. Genetic susceptibility involving polymorphisms in SCN9A, encoding the Nav1.7 sodium channel, and catechol-O-methyltransferase (COMT) has been associated with altered pain sensitivity and increased vulnerability to chronic pain states [17]. Furthermore, enhanced α-amino-3-hydroxy-5-methyl-4-isoxazolepropionic acid (AMPA) receptor trafficking, excessive glutamate release, and astrocytic gliotransmitter signaling further reinforce excitatory synaptic transmission, ultimately promoting neuronal hyperexcitability, long-term potentiation, and central sensitization [18]. This cascade ultimately induces persistent activation of pain-related circuits and contributes to maladaptive neuroplasticity and structural and functional remodeling across multiple pain-related brain regions [19]. For example, chronic pain has been associated with reductions in gray matter volume in regions such as the anterior cingulate cortex (ACC), superior and middle frontal gyri, and other corticolimbic structures, together with enhanced functional connectivity involving medial prefrontal cortex–nucleus accumbens circuits and altered interactions among the default mode, salience, and central executive network [20]. Clinically, these network-level alterations contribute to pain-related emotional and cognitive dysfunction [21].

Concurrent dysregulation of the hypothalamic–pituitary–adrenal axis, chronic cortisol imbalance, anxiety, depression, and social stressors may further amplify these maladaptive feedback mechanisms [22]. Importantly, these mechanisms may be particularly relevant during childhood and adolescence, when synaptic pruning, myelination, and large-scale functional network maturation are still ongoing [23]. Persistent nociceptive input during these developmental stages may interfere with normal neurodevelopmental trajectories, potentially resulting in long-lasting alterations in salience processing, emotional regulation, executive functioning, and pain vulnerability across the lifespan [5].

Collectively, these molecular, cellular, and neuroimmune mechanisms may represent the biological substrate through which peripheral and central sensitization evolve into large-scale alterations of brain function. Persistent nociceptive signaling, maladaptive neuroplasticity, and chronic neuroinflammation are increasingly thought to contribute not only to local circuit remodeling but also to disruptions in communication among distributed neural systems. From this perspective, the transition from acute to chronic pain can be viewed as a multiscale process linking microscopic changes in neuronal and glial function with macroscopic alterations in functional connectivity with large-scale network organization [24].

## 4. Strengths and Pitfalls of the Pain Matrix

Historically, neuroimaging studies have conceptualized pain-related brain activity in terms of a so-called pain matrix. The term encompasses a set of cortical and subcortical regions consistently activated during experimental pain. Neuroimaging and neurophysiological investigations have identified multiple brain regions that reliably respond to nociceptive stimulation across different experimental paradigms [21,25,26,27]. This evidence supports the view that pain emerges from the integration of multiple functional domains rather than from a single cortical locus. These regions contribute in distinct yet interacting ways to the sensory, cognitive, and affective dimensions of pain experience. Within this distributed system, the thalamus serves as a critical hub, relaying and modulating nociceptive information from peripheral and spinal pathways to cortical targets. Cortical processing of pain prominently involves the insular cortex, with the posterior and mid-insular subdivisions playing a central role in encoding the intensity and thermal qualities of painful stimuli. Notably, insular activation is observed in both acute and chronic pain states, suggesting its involvement in the sustained representation and modulation of pain over time [26].

The primary and secondary somatosensory cortices (S1 and S2) are principally engaged in the sensory-discriminative components of pain, supporting spatial localization and intensity coding. In parallel, affective and motivational aspects of pain are strongly associated with the anterior cingulate cortex, which contributes to the emotional salience of pain and to behavioral responses aimed at avoidance or coping. Higher-order cognitive appraisal and regulation of pain involve prefrontal cortical regions, particularly the dorsolateral prefrontal cortex, which are implicated in attentional control, decision-making, and the contextual evaluation of pain-related information.

Beyond these cortical nodes, subcortical and brainstem structures play a pivotal modulatory role. The periaqueductal gray within the midbrain constitutes a key component of descending pain control systems, mediating endogenous analgesia and adaptive regulation of pain sensitivity. Limbic structures, including the nucleus accumbens and amygdala, link pain processing to motivational and emotional states, influencing reward learning, aversion, and pain-related memory. The hippocampus further contributes to the contextual and mnemonic dimensions of pain, while the cerebellum has been increasingly recognized for its role in integrating sensorimotor and affective information related to painful experiences [27]. Collectively, these interconnected regions form a dynamic and distributed network that supports the multifaceted nature of pain. Therefore, pain processing arises from the coordinated activity of sensory, emotional, cognitive, and modulatory systems, which ultimately shape individual pain perception and behavioral responses.

Nevertheless, this framework has been criticized. For example, the lack of specificity of many pain matrix regions, which are also engaged by non-painful salient stimuli, contrasts with the notion of a dedicated and modular pain system [27,28]. Even before the emergence of contemporary network neuroscience, Melzack’s neuromatrix theory had already proposed that pain arises from the dynamic integration of distributed sensory, affective, cognitive, and evaluative processes rather than from the activation of a dedicated nociceptive center [8]. Current network-based models may be viewed as a neurobiological extension of this conceptual framework. From this perspective, a growing body of experimental evidence has questioned the classical interpretation of the pain matrix as a direct neural representation of pain experience [28,29]. Several studies have shown that subjective pain intensity does not necessarily scale with the amplitude of neural responses in regions traditionally ascribed to the pain matrix [29]. Rather than reflecting pain per se, these neural responses appear highly sensitive to contextual factors, including stimulus novelty, expectancy, and attentional engagement. Converging findings from laser-evoked electroencephalography (EEG) and functional magnetic resonance imaging (fMRI) further support this view. Specifically, cortical activation patterns resembling those attributed to the pain matrix can be elicited by non-nociceptive stimuli or by stimuli not consciously perceived as painful, indicating a lack of specificity for pain processing [30]. Although correlations between brain activity and pain perception are frequently reported, such associations do not establish a causal relationship between neuronal activation and the subjective experience of pain [28]. This dissociation is particularly evident in individuals with congenital insensitivity to pain, who exhibit activation patterns within so-called pain matrix regions comparable in magnitude to those observed in pain-sensitive controls, despite the absence of pain perception [31]. Moreover, reverse inference remains a major limitation of functional neuroimaging, as activation of a given brain region cannot be assumed to reflect a specific psychological process in the absence of convergent experimental evidence [32]. Concerns regarding reproducibility and variability across imaging protocols further highlight the need for cautious interpretation of putative neuroimaging biomarkers [33]. These observations underscore the limitations of a purely structure–function interpretation of pain processing based on regional activation. Independent of the pain matrix framework, a central challenge remains to elucidate how nociceptive sensory input is transformed into the conscious experience of pain, and how inflammatory processes, tissue injury, or sustained nociceptive signaling give rise to peripheral and central sensitization. These mechanisms, in turn, drive enduring neuroplastic changes that shape the clinical expression, persistence, and heterogeneity of chronic pain syndromes [34].

Using fMRI techniques in newborns, Goksan et al. [35] showed that most of the same pain matrix areas as in adults (somatosensory cortex, insula, ACC, thalamus) are also activated after nociceptive stimuli in infants. Of 20 regions activated in adults, 18 showed significant activity in newborns subjected to the same painful stimulus. This suggests that the developing brain already possesses the sensory and affective networks involved in adult pain. These studies highlight the importance of adequate analgesic strategies in children; at the same time, they confirm that even in pediatric age, the so-called pain matrix has a functional basis similar to that of adults.

## 5. Pain-Related Functional Network

The correlations between key networks and clinical manifestation represent a research challenge of pivotal importance. Therefore, researchers suggested a conceptual shift from region-based models toward network-oriented perspectives. Chronic pain is now increasingly understood as a disorder of large-scale brain networks, involving dysfunctional interactions among the salience network (SN), default mode network (DMN), central executive network (CEN), and sensorimotor systems [6,7,8].

Connectivity between the medial prefrontal cortex (mPFC) and the anterior insula (SN) is increased in subjects with chronic pain [36]. Beyond its role as a major node of the DMN, the mPFC may function as an integrative hub linking nociceptive processing with affective regulation, stress responsiveness, and top-down cognitive modulation. Rather than representing an isolated biomarker, mPFC dysfunction should be interpreted within a system-level framework reflecting large-scale network reorganization in chronic pain [37].

Rather than representing isolated regional abnormalities, chronic pain appears to involve widespread alterations in intrinsic brain organization and network communication. This concept has been further reinforced by a recent systematic review and meta-analysis of resting-state fMRI studies conducted by Fiúza-Fernandes et al. [36]. Integrating evidence from multiple analytical approaches, the authors identified convergent abnormalities in spontaneous neural activity and functional connectivity involving key regions such as the precuneus, medial prefrontal cortex, and insula. Specifically, significant alterations were observed in the amplitude of low-frequency fluctuations (ALFF) and fractional amplitude of low-frequency fluctuations (fALFF), which reflect spontaneous regional brain activity, as well as in regional homogeneity (ReHo), an index of local synchronization of neural activity. Consistent findings were also reported using seed-based functional connectivity and independent component analyses. Collectively, these results support the conceptualization of chronic pain as a disorder of altered intrinsic brain function, characterized by disruption of major DMN hubs, salience-processing regions, and top-down pain modulatory systems [36]. Such large-scale network dysfunction may contribute to the persistence of pain, maladaptive attentional allocation, emotional dysregulation, and impaired cognitive control.

From a predictive coding perspective, chronic pain may be interpreted as a maladaptive perceptual state in which prior expectations and pain-related predictions progressively exert a stronger influence on perception than incoming sensory evidence [38]. Within this Bayesian framework [39], maladaptive updating of prediction errors and the progressive stabilization of pain-related priors may contribute to the persistence and amplification of pain experiences. Experimentally, these mechanisms have been investigated through expectancy manipulation paradigms such as placebo/nocebo models [40].

The self-representational DMN is a functional network that is active during rest. It is composed of the ventromedial PFC, posterior cingulate cortex (PCC), inferior parietal cortex (IPC), pregenual ACC, and precuneus and is involved in self-referential thoughts, daydreaming, and mind-wandering. DMN plays a role in introspection, mentation processes, memory retrieval, and planning. In individuals with chronic pain, DMN often shows disrupted connectivity [41]. These changes may favor persistent pain-related rumination and increased self-referential attention to symptoms, potentially contributing to the maintenance of chronic pain states. Furthermore, evidence suggests that DMN abnormalities may become more pronounced with increasing duration of pain, particularly in chronic low back pain populations [42]. Beyond conventional spectral analyses, emerging studies on EEG microstates suggest that transient large-scale electrophysiological configurations may provide dynamic biomarkers of salience processing, cognitive–emotional integration, and treatment responsiveness in chronic pain [43]. These findings offer a complementary electrophysiological perspective on the functional network alterations identified through neuroimaging studies. Importantly, alterations of the DMN are not restricted to chronic pain states and have also been observed during acute pain processing [44].

**Figure 1:**
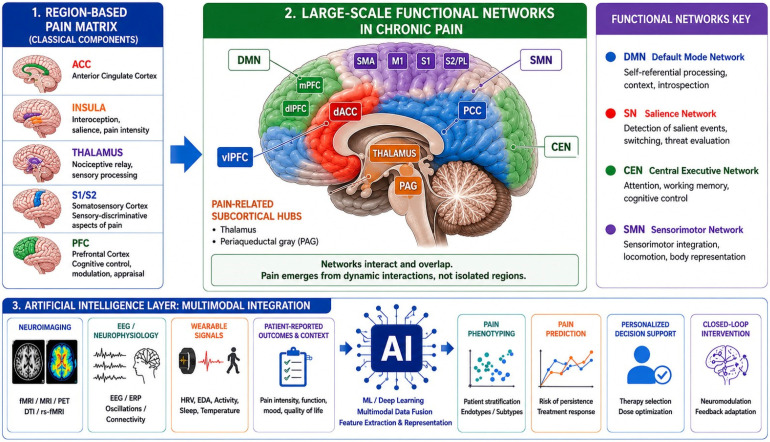
Evolution from region-based pain matrix models to large-scale functional brain networks and artificial intelligence-driven multimodal integration in chronic pain. Transition from classical region-based pain matrix components to distributed functional network organization and, subsequently, to artificial intelligence (AI)-based multimodal integration. The left panel summarizes key anatomical regions traditionally associated with pain processing, including the anterior cingulate cortex, insula, thalamus, somatosensory cortices, and prefrontal cortex. The central panel shows large-scale functional networks involved in chronic pain, highlighting the spatial overlap and interaction between the default mode, salience, central executive, and sensorimotor networks, together with pain-related subcortical hubs. The lower panel illustrates how neuroimaging, electrophysiological, wearable-derived, and patient-reported data can be integrated through AI-based models to support pain phenotyping, prediction, personalized decision support, and closed-loop interventions. Abbreviations: ACC, anterior cingulate cortex; AI, artificial intelligence; AU, action unit; CEN, central executive network; dACC, dorsal anterior cingulate cortex; dlPFC, dorsolateral prefrontal cortex; DMN, default mode network; DTI, diffusion tensor imaging; EDA, electrodermal activity; EEG, electroencephalography; ERP, event-related potential; fMRI, functional magnetic resonance imaging; HRV, heart rate variability; M1, primary motor cortex; mPFC, medial prefrontal cortex; MRI, magnetic resonance imaging; PAG, periaqueductal gray; PCC, posterior cingulate cortex; PET, positron emission tomography; PFC, prefrontal cortex; PL, parietal lobule; PROs, patient-reported outcomes; rs-fMRI, resting-state functional magnetic resonance imaging; S1, primary somatosensory cortex; S2, secondary somatosensory cortex; SMA, supplementary motor area; SMN, sensorimotor network; SN, salience network; vlPFC, ventrolateral prefrontal cortex.

Another major large-scale network implicated in pain-related brain function is the SN. This network is involved in identifying and filtering behaviorally relevant stimuli, allocating attentional resources, and facilitating adaptive responses to environmental demands. Its principal nodes include the anterior insular cortex and the dorsal ACC. Functional neuroimaging studies have reported abnormal SN activity and connectivity in several chronic pain conditions. Such alterations may increase the salience attributed to nociceptive information, thereby amplifying pain perception and potentially contributing to mechanisms of central sensitization. Nevertheless, the direction and magnitude of these changes vary across studies [43]. Given SN functioning, altered SN activity and connectivity may influence pain salience attribution, attentional bias toward nociceptive information, and the integration of sensory and affective components of pain. Such mechanisms have been proposed to contribute to pain chronification and maladaptive network reorganization [41,44,45]. The SN maintains close functional interactions with the CEN, a goal-directed frontoparietal system involved in cognitive control, whereas both networks typically exhibit an inverse relationship with the DMN [38]. Within this framework, pain persistence is associated not simply with increased activation of nociceptive regions, but with altered network balance, impaired switching between internally and externally oriented states, and aberrant integration of sensory signals with self-referential and affective processing. Figure 1 further translates these concepts into an integrated visual representation of the overlapping neural systems and multimodal analytical layers that may support future precision pain medicine.

These network-level alterations provide a more coherent account of key clinical features of chronic pain, including symptom persistence, comorbid affective disturbances, cognitive impairment, and interindividual variability in pain experience and treatment response [13]. Importantly, these network interactions may also help explain the dissociation between nociceptive intensity and subjective suffering. While sensory-discriminative pathways contribute to the localization and intensity of pain, higher-order salience, limbic, and executive systems shape the perceived threat, emotional burden, and behavioral significance of pain [37].

From a multiscale perspective, alterations in functional connectivity represent the macroscopic expression of cellular, synaptic, and molecular changes occurring within pain-related circuits, thereby bridging network dysfunction with the biological substrates of pain chronification. Chronic pain is associated with widespread neuroanatomical and molecular alterations involving mesolimbic and mesocortical circuits, maladaptive neuroplasticity, dysregulated dopaminergic signaling, and structural remodeling of pain-related brain regions, including the prefrontal cortex, amygdala, hippocampus, and nucleus accumbens. Alterations in the mesocortical pathway in conditions of chronic pain involve, for example, an increase in dendritic branching and a reduction in gray matter. Additionally, chronic pain is associated with alterations in mesolimbic dopaminergic signaling, reduced neuroplasticity, and impaired neurogenesis, processes in which brain-derived neurotrophic factor (BDNF) appears to play a central role [46,47]. Chronic pain states are also characterized by neuroinflammatory processes involving increased levels of pro-inflammatory cytokines such as IL-6 and TNF-α, glial activation, altered excitatory and inhibitory synaptic transmission, and dysregulation of the mechanistic target of rapamycin complex 1 (mTORC1) pathway, all of which contribute to maladaptive cortical plasticity and cognitive dysfunction [48,49,50].

Targeting mTORC1 signaling in spinal cord neurons may provide a new strategy for pain management in neuropathic pain [51]. Inhibiting mTOR signaling in the insular cortex reduces neuropathic pain after peripheral nerve injury by inhibiting synaptic plasticity and reducing mechanical allodynia [52].

Together, these mechanisms may contribute to a vicious cycle linking chronic pain and cognitive deficits, highlighting the complex interaction between nociceptive and cognitive processes and emphasizing the need for targeted therapeutic interventions.

Similar abnormalities in pain-related functional networks have also been observed in children and adolescents. For example, Jones et al. [53] showed that in adolescents, pain frequency is associated with changes in DMN connectivity: the more frequent the painful episodes, the greater the connectivity between the DMN and the higher prefrontal cortex, while higher pain intensity is linked to reduced connectivity between the DMN and the cerebellum. This indicates that, already at a young age, a brain exposed to persistent pain shows DMN disruption and altered connections with cognitive areas. A pivotal study by Becerra et al. [54] on children with complex regional pain syndrome (CRPS) confirmed that multiple networks are compromised: in the acute phase, young patients showed abnormalities in the salience network, the DMN, the central executive network (fronto-parietal), and the sensorimotor network, compared to healthy peers. Despite improved clinical outcomes in many pediatric patients, fMRI analyses have shown that intensive physical and psychological treatment may reduce aberrant connectivity within the SN, DMN, CEN, and sensorimotor networks [54]. It should be noted that the pediatric neuroimaging literature remains substantially smaller than the corresponding adult literature, with most available studies involving relatively limited sample sizes and predominantly cross-sectional designs [35,53,54]. Consequently, developmental and causal inferences remain constrained, and further longitudinal investigations are needed to better characterize the evolution of pain-related network alterations across childhood and adolescence. Preliminary evidence suggests that pediatric chronic pain may involve partially overlapping network alterations with those reported in adults; however, this interpretation should be considered tentative given the limited pediatric evidence base [35,53,54]. Nevertheless, current findings indicate that some pain-related network abnormalities may be at least partially reversible following appropriate therapeutic intervention [54]. Importantly, these large-scale network alterations are not only mechanistic descriptors of chronic pain but may also provide measurable targets for computational analysis. Functional connectivity patterns within the DMN, SN, CEN, and sensorimotor systems generate multidimensional signatures that can be integrated with behavioral, autonomic, electrophysiological, and neuroimaging data. In this context, AI methodologies may help identify latent network phenotypes, characterize individual pain trajectories, and support personalized therapeutic strategies [55,56]. Thus, AI should be viewed not as a separate technological domain, but as a potential analytical framework for translating network-level neuroscience into clinically actionable pain biomarkers [10,57].

## 6. Research Perspectives and AI Applications

Despite the conceptual advances offered by network-based models, several limitations constrain their direct translation into clinical decision-making. While large-scale functional networks provide a compelling framework for explaining pain persistence, affective comorbidities, and cognitive dysfunction, they currently lack sufficient specificity to guide individualized treatment selection. In particular, network alterations alone do not readily translate into actionable therapeutic targets, nor do they adequately account for the heterogeneity of clinical trajectories observed in patients with chronic pain [21,58].

This gap between mechanistic insight and therapeutic applicability has contributed, at least in part, to an overreliance on symptom-oriented pharmacological strategies. In parallel with the rising prevalence of chronic pain, the widespread use of opioid therapy has been recognized as a major contributor to the opioid epidemic [59,60]. Consequently, greater emphasis has been placed on physician education, opioid stewardship, risk evaluation and mitigation strategies (REMS), prescription drug monitoring programs (PDMPs), and the search for personalized, mechanism-informed approaches capable of translating advances in pain neuroscience into clinical practice [60,61]. These concerns have accelerated interest in non-opioid and multimodal approaches to chronic pain management. Behavioral and psychosocial interventions, such as Mindfulness-Oriented Recovery Enhancement (MORE), have demonstrated potential in addressing not only pain intensity but also the emotional suffering, maladaptive coping, and reward dysregulation that accompany chronic pain [62]. Such approaches resonate with network-based models by targeting cognitive–affective dimensions of pain that are poorly addressed by pharmacological analgesia alone.

Notably, as emphasized by Ling et al. [63], opioids should be regarded as one component within a broader therapeutic landscape rather than as a central or definitive solution for chronic pain. From this perspective, effective pain management should prioritize interventions aimed at alleviating suffering, restoring function, and improving quality of life, rather than focusing exclusively on nociceptive suppression [60,64].

Against this background, the development of approaches that can bridge mechanistic models with individualized therapy selection represents a major unmet need. AI offers a potential pathway to address this challenge by integrating clinical profiles, network-level biomarkers, and longitudinal outcomes to support data-driven, personalized pain management strategies.

In recent years, several AI applications have been proposed for addressing chronic pain assessment, phenotyping, and treatment optimization [65]. Current applications of AI in the field of chronic pain aim to extract meaningful representations from complex data. The goals of personalized medicine are based on identifying patient subgroups, refining therapeutic approaches, identify patterns, correlations, and biomarkers associated with specific pain profiles [65].

Specifically, the analysis of structured clinical datasets from well-characterized patient populations enables the identification of pain subtypes, supports stratified clinical decision-making, and allows the prediction of disease trajectories, treatment response, and risk of adverse outcomes. In this framework, AI may contribute to the early identification of individuals at higher risk of pain persistence or complications, thereby informing timely and targeted interventions. Nevertheless, the clinical translation of AI-based approaches is challenged by the intrinsic complexity of pain physiology, limited generalizability across heterogeneous patient populations, and variability in data quality and completeness [10,66]. At present, most digital biomarkers proposed for chronic pain should be considered exploratory physiological correlates rather than fully validated clinical biomarkers, pending external validation, reproducibility studies, and demonstration of clinical utility [11,45].

An additional methodological challenge is the lack of a robust ground truth for pain, as pain remains a multidimensional, subjective, and context-dependent experience that cannot be fully captured through single labels, surrogate outcomes, or symptom-based classifications alone. Progress toward clinically meaningful AI will therefore require biologically informed phenotyping and more precise multimodal target definitions.

**Figure 2:**
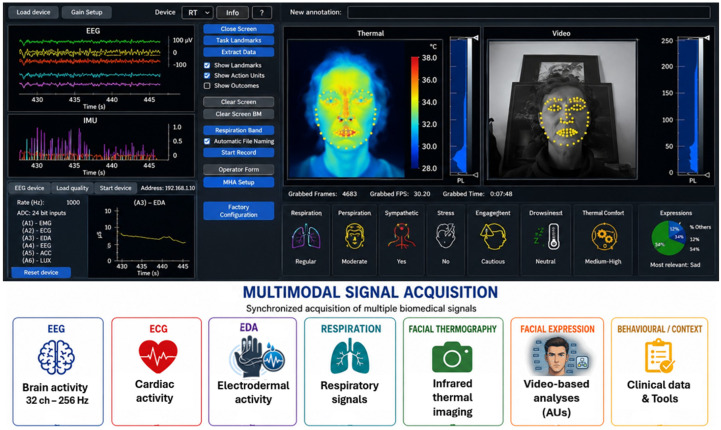
Hybrid InfraRed Affective computing (HIRA) system for multimodal acquisition of facial thermography, visual expressions, and physiological biosignals in AI-driven pain research. The figure illustrates the HIRA (Hybrid InfraRed Affective computing) platform developed as part of the authors’ Refining Multiple Artificial Intelligence Strategies for Automatic Pain Assessment Investigations (RUGGI) project. The system integrates infrared facial thermography, visual facial expression analysis, facial landmark tracking, and synchronized physiological biosignals, including electroencephalography (EEG), electrocardiography (ECG), galvanic skin response (GSR), and respiratory signals. This multimodal framework was designed to characterize autonomic, neurophysiological, and affective pain-related signatures by continuously extracting thermal, visual, and biosignal-derived digital biomarkers. The platform is presented as a conceptual and translational research framework and should not be interpreted as an independently validated clinical system. Clinical validation studies are ongoing. Framework from the Refining Multiple Artificial Intelligence Strategies for Automatic Pain Assessment Investigations (RUGGI) Project (ClinicalTrials.gov identifier: NCT07038434).

Despite these limitations, accumulating evidence indicates that AI already provides tangible support in selected areas of pain medicine. For instance, automated analysis of facial expressions has been shown to assist in categorizing pain perception, offering an objective complement to self-reported measures [67,68,69]. Similarly, wearable and sensor-based technologies, including electrodermal activity monitoring, have facilitated more objective assessments of pain-related autonomic responses, further expanding the toolkit available for pain evaluation [70]. As an example of a translational multimodal framework currently under investigation, Figure 2 illustrates the Hybrid InfraRed Affective computing (HIRA) system developed within the authors’ RUGGI project [1,71]. The platform was designed to integrate facial thermography, visual expression analysis, and physiological biosignals for research purposes in AI-assisted pain assessment. At its current stage, HIRA should be considered a conceptual and research-oriented framework rather than an independently validated clinical system [72]. However, the translational adoption of multimodal AI platforms remains constrained by several methodological challenges, including dataset shift across institutions and devices, hidden confounding, non-trivial risks of data leakage within multimodal pipelines, and limited external validation across heterogeneous patient populations.

The need for such advances is underscored by epidemiological data showing that a substantial proportion of patients with chronic pain continue to experience persistent symptoms despite prolonged treatment. According to Nahin et al. [73], most individuals with chronic pain report ongoing pain even after one year of care, highlighting the inadequacy of uniform treatment strategies and reinforcing the importance of patient-centered approaches, an area in which AI may play a pivotal role.

Beyond clinical interviews and patient counseling, pain assessment can rely on experimental and instrumental techniques, including quantitative sensory testing, electrophysiological recordings, and neuroimaging measures of nociceptive processing. These methods are particularly suited to the study of evoked pain phenomena, such as hyperalgesia and allodynia, as they involve controlled painful stimulation. In contrast, spontaneous pain, arguably the most clinically relevant dimension of chronic pain, is better captured through functional neuroimaging and neurophysiological approaches, including EEG, which allow the investigation of ongoing brain activity without external stimulation [43].

AI methodologies widely adopted in healthcare include ML and deep learning (DL) techniques. ML algorithms can process electronic health records, medical images, and multidimensional clinical data to support diagnosis, predict outcomes, and personalize treatments [74]. DL, a subset of AI based on artificial neural networks, is particularly effective in handling complex, high-dimensional data and has proven valuable in image and speech recognition tasks relevant to medical applications [75,76]. The progressive refinement of these technologies represents both a major opportunity and a substantial challenge for their integration into routine clinical practice.

Importantly, AI-driven innovations may contribute to reshaping pain management strategies, with the potential to improve clinical outcomes, enhance quality of life, and reduce reliance on opioid-based therapies. However, significant knowledge gaps remain. One critical area for future research is the limited integration of AI with real-time physiological feedback systems. Large-scale, multimodal studies are needed to evaluate how continuous physiological inputs can be combined with adaptive algorithms to inform dynamic treatment modulation [74].

In this context, AI-assisted analysis of neuroimaging data offers promising opportunities to elucidate how interventions such as spinal cord stimulation influence brain activity and pain perception. Techniques such as functional magnetic resonance imaging and EEG have already demonstrated value in characterizing neural correlates of pain, but the sheer volume and complexity of these data limit traditional analytical approaches [77,78]. DL algorithms may therefore be essential to identify clinically meaningful patterns associated with effective pain relief and to support adaptive neuromodulation strategies [74].

Consistent with this vision, recent studies suggest that associating specific neural activity patterns with reductions in pain perception could enable automated adjustment of stimulation parameters, thereby facilitating more effective and individualized treatment plans [79,80]. A representative example is the EcoAI platform, which integrates transcutaneous electrical nerve stimulation (TENS) and neuromuscular electrical stimulation (NMES) with AI-driven customization and remote assistance. Unlike conventional devices, EcoAI continuously adapts stimulation parameters, such as frequency, amplitude, and pulse duration, in real time based on individual patient profiles, combining subjective pain reports with objective physiological indicators, including heart rate variability and muscle activity. This closed-loop system exemplifies how AI can allow for individualized neuromodulation through continuous sensing, analysis, and feedback [81]. Similarly, non-invasive neuromodulation approaches such as transcranial direct current stimulation (tDCS) are gaining attention, particularly in oncology, where preliminary evidence suggests benefits not only on pain intensity but also on cognitive and affective symptoms [82,83].

Beyond neuromodulation, AI-based cognitive-behavioral therapy (CBT) programs represent another emerging application, in which adaptive algorithms learn from large-scale patient interactions over time, progressively refining therapeutic content and delivery to enhance effectiveness [84].

Overall, moving beyond region-based representations of the pain matrix toward integrated network models, and cautiously embedding AI within this framework, offers a path to reconcile mechanistic understanding with clinical complexity, ultimately enabling more precise, personalized, and clinically meaningful approaches to chronic pain. Nevertheless, AI systems should be viewed as decision support tools designed to augment, rather than replace, clinical judgment and the inherently subjective assessment of pain performed by healthcare professionals [58].

To summarize the current state of evidence across mechanistic, developmental, and translational domains, Table 1 provides an evidence-based mapping of major research areas in chronic pain neuroscience and emerging AI applications.

To synthesize this conceptual evolution, Table 2 outlines the major theoretical, biological, and clinical transitions from regional models to network-based and AI-enhanced frameworks.

## 7. Conclusions

Chronic pain is increasingly recognized as a multiscale and dynamic disorder that cannot be fully explained by isolated nociceptive pathways or by regional patterns of brain activation alone. While the pain matrix framework has historically provided important insights into the sensory, affective, and cognitive dimensions of pain processing, accumulating evidence indicates that pain persistence is more accurately understood as the consequence of maladaptive interactions among large-scale functional networks, including the default mode, salience, executive control, and sensorimotor systems. This conceptual transition is particularly relevant when adopting a lifespan perspective. In pediatric populations, persistent pain may interact with ongoing neurodevelopmental processes, potentially influencing network maturation, emotional regulation, cognitive performance, and long-term behavioral adaptation. Conversely, in adult patients, prolonged pain may reinforce maladaptive neuroplasticity, functional disconnection, and affective–cognitive dysfunction, contributing to clinical heterogeneity and variable treatment responsiveness.

Despite substantial advances in network neuroscience, translating these mechanistic insights into individualized therapeutic strategies remains a major clinical challenge. In this context, AI offers a promising opportunity to bridge the gap between biological complexity and precision medicine. By integrating multimodal information derived from neuroimaging, electrophysiology, wearable biosensors, digital biomarkers, and patient-reported outcomes, AI-based approaches may enable objective pain phenotyping, early risk stratification, prediction of clinical trajectories, and adaptive treatment optimization.

Nevertheless, significant barriers remain, including limited external validation, heterogeneity of data acquisition protocols, lack of explainability, and insufficient integration into real-world clinical workflows. Additional barriers include regulatory approval requirements, reimbursement frameworks, workflow integration, and clinician acceptance, all of which may influence large-scale clinical implementation. Future research should prioritize large-scale longitudinal studies, multimodal harmonization, pediatric-inclusive datasets, and human-centered AI frameworks and adaptive closed-loop neuromodulation systems for integrating continuous physiological feedback into personalized pain management strategies.

Ultimately, moving from region-based representations toward integrated network models, while cautiously embedding artificial intelligence within this framework, may help redefine chronic pain as a measurable, biologically informed, and increasingly personalized clinical condition.

## Figures and Tables

**Table 1:** Evidence map of major mechanistic and translational domains in chronic pain research across adult and pediatric populations.

Domain	Representative Evidence	Main Findings	Clinical Relevance	Current Limitations	Future Directions
Pain matrix and regional models	Experimental neuroimaging, lesion studies, EEG/fMRI investigations	Consistent activation of insula, ACC, thalamus, S1/S2, and PAG during nociceptive stimulation	Improved understanding of sensory-discriminative and affective dimensions of pain	Limited specificity; overlap with salience and attention processing	Integration with network-level and causal models
Large-scale functional networks	Resting-state fMRI, connectivity meta-analyses	Altered connectivity within DMN, SN, CEN, and sensorimotor networks in chronic pain	Explains symptom persistence, cognitive dysfunction, and emotional comorbidities	Limited individual-level specificity; cross-sectional predominance	Longitudinal connectomics and individualized network phenotyping
Predictive coding and cognitive–affective mechanisms	Computational neuroscience, behavioral, and neuroimaging studies	Pain persistence may reflect maladaptive priors, salience amplification, and impaired sensory updating	Provides a mechanistic explanation for pain–emotion interactions and catastrophizing	Difficult clinical operationalization; limited biomarkers	Integration with computational psychiatry and digital phenotyping
Molecular and cellular neuroplasticity	Preclinical studies, translational neuroscience	Altered dopaminergic signaling, BDNF modulation, glial activation, neuroinflammation, mTOR dysregulation	Identifies potential therapeutic targets for neuromodulation and pharmacological interventions	Limited direct translation from animal models to humans	Multiscale biomarker integration with human neuroimaging
Pediatric chronic pain and neurodevelopment	Developmental neuroimaging, longitudinal pediatric cohorts	Altered network maturation, emotional regulation, and cognitive development	Supports early intervention and developmental precision medicine	Small cohorts; limited longitudinal data	Age-adaptive biomarkers and developmental network modeling
Electrophysiological biomarkers	EEG, microstate analysis, spectral connectivity studies	Abnormal oscillatory patterns and altered microstate dynamics in chronic pain	Potential for low-cost, real-time pain monitoring	Protocol heterogeneity; limited Standardization	Closed-loop EEG-guided interventions
Wearable and autonomic biomarkers	EDA, HRV, multimodal biosignal studies	Autonomic alterations may correlate with pain episodes and treatment response	Enables continuous ecological monitoring	Motion artifacts, signal variability, and incomplete contextualization	Digital biomarkers integrated with mobile health ecosystems
Artificial intelligence applications	ML/DL studies using clinical, imaging, and biosignal datasets	AI supports pain phenotyping, outcome prediction, facial expression analysis, and multimodal classification	Potential for personalized treatment selection and early risk stratification	Dataset bias, limited external validation, and explainability concerns	Federated learning, explainable AI, multimodal longitudinal models
AI-driven neuromodulation and closed-loop systems	Pilot studies in SCS, TENS, NMES, and EEG-guided stimulation	Adaptive stimulation based on physiological feedback may improve personalization	Supports real-time treatment optimization	Early-stage evidence; regulatory and interoperability challenges	Fully adaptive closed-loop precision pain platforms

Abbreviations: AI, artificial intelligence; ACC, anterior cingulate cortex; BDNF, brain-derived neurotrophic factor; CEN, central executive network; DMN, default mode network; EDA, electrodermal activity; EEG, electroencephalography; HRV, heart rate variability; ML, machine learning; NMES, neuromuscular electrical stimulation; PAG, periaqueductal gray; SCS, spinal cord stimulation; SN, salience network; TENS, transcutaneous electrical nerve stimulation.

**Table 2:** Conceptual evolution of chronic pain models: from pain matrix to network neuroscience and artificial intelligence-driven precision pain medicine.

Conceptual Domain	Pain Matrix Model	Network-Based Model	AI-Enhanced Precision Model
Theoretical framework	Region-based representation of pain-related brain activation	Distributed interactions among large-scale functional networks	Data-driven integration of neurobiological, behavioral, and clinical information
Main biological focus	Cortical and subcortical activation (e.g., insula, ACC, thalamus)	DMN, SN, CEN, sensorimotor connectivity and network dynamics	Multimodal biomarkers, digital phenotypes, longitudinal trajectories
Interpretation of pain	Response to nociceptive stimuli	Emergent property of network dysregulation and maladaptive neuroplasticity	Individualized pain signatures and predictive phenotyping
Clinical relevance	Improved mechanistic understanding	Explains cognitive dysfunction, emotional comorbidities, and symptom persistence	Supports risk prediction, treatment selection, and adaptive interventions
Pediatric implications	Limited developmental specificity	Accounts for network maturation and developmental plasticity	Potential for early phenotyping and personalized developmental interventions
Major limitations	Limited specificity, overlap with salience processing	Limited direct therapeutic translation	Need for validation, explainability, ethical governance, interoperability
Future perspectives	Historical conceptual foundation	Systems-level mechanistic modeling	Real-time precision pain medicine and closed-loop therapeutics

Abbreviations: AI, artificial intelligence; ACC, anterior cingulate cortex; CEN, central executive network; DMN, default mode network; SN, salience network.

## Data Availability

No new data were created or analyzed in this study.

## References

[1] Cascella M., Guerra C., De Feo R., Di Lisio F., Giordano P., Esposito W., Cisale G., Cerrone V., Esposito D., Bruno M.P. (2026). A Prospective Multimethod Investigation of Cancer-Related Pain Integrating Clinical Data and Machine Learning: Results from the RUGGI Study. J. Anesth. Analg. Crit. Care.

[2] Delivering Transformative Action in Paediatric Pain: A Lancet Child & Adolescent Health Commission—The Lancet Child & Adolescent Health. https://www.thelancet.com/journals/lanchi/article/PIIS2352-4642(20)30277-7/fulltext.

[3] Chambers C.T., Dol J., Tutelman P.R., Langley C.L., Parker J.A., Cormier B.T., Macfarlane G.J., Jones G.T., Chapman D., Proudfoot N. (2024). The Prevalence of Chronic Pain in Children and Adolescents: A Systematic Review Update and Meta-Analysis. Pain.

[4] Palermo T.M. (2020). Pain Prevention and Management Must Begin in Childhood: The Key Role of Psychological Interventions. Pain.

[5] Bhatt R.R., Gupta A., Mayer E.A., Zeltzer L.K. (2020). Chronic Pain in Children: Structural and Resting-State Functional Brain Imaging within a Developmental Perspective. Pediatr. Res..

[6] Zhang S., Ning Y., Yang Y., Mu G., Yang Y., Ren C., Liao C., Ou C., Zhang Y. (2025). Decoding Pain Chronification: Mechanisms of the Acute-to-Chronic Transition. Front. Mol. Neurosci..

[7] Cascella M., Muzio M.R., Monaco F., Nocerino D., Ottaiano A., Perri F., Innamorato M.A. (2022). Pathophysiology of Nociception and Rare Genetic Disorders with Increased Pain Threshold or Pain Insensitivity. Pathophysiology.

[8] Melzack R. (2005). Evolution of the Neuromatrix Theory of Pain. The Prithvi Raj Lecture: Presented at the Third World Congress of World Institute of Pain, Barcelona 2004. Pain Pract..

[9] De Ridder D., Vanneste S., Smith M., Adhia D. (2022). Pain and the Triple Network Model. Front. Neurol..

[10] Casarin S., Haelterman N.A., Machol K. (2024). Transforming Personalized Chronic Pain Management with Artificial Intelligence: A Commentary on the Current Landscape and Future Directions. Exp. Neurol..

[11] Mackey S., Aghaeepour N., Gaudilliere B., Kao M.-C., Kaptan M., Lannon E., Pfyffer D., Weber K. (2025). Innovations in Acute and Chronic Pain Biomarkers: Enhancing Diagnosis and Personalized Therapy. Reg. Anesth. Pain Med..

[12] Baethge C., Goldbeck-Wood S., Mertens S. (2019). SANRA—A Scale for the Quality Assessment of Narrative Review Articles. Res. Integr. Peer Rev..

[13] Song Q., E S., Zhang Z., Liang Y. (2024). Neuroplasticity in the Transition from Acute to Chronic Pain. Neurotherapeutics.

[14] Schumacher M.A. (2024). Peripheral Neuroinflammation and Pain: How Acute Pain Becomes Chronic. Curr. Neuropharmacol..

[15] Liu B.-W., Zhang J., Hong Y.-S., Li N.-B., Liu Y., Zhang M., Wu W.-Y., Zheng H., Lampert A., Zhang X.-W. (2021). NGF-Induced Nav1.7 Upregulation Contributes to Chronic Post-Surgical Pain by Activating SGK1-Dependent Nedd4-2 Phosphorylation. Mol. Neurobiol..

[16] Sun J., Lu L., Lian Y., Xu S., Zhu Y., Wu Y., Lin Q., Hou J., Li Y., Yu Z. (2025). Sodium Butyrate Attenuates Microglia-Mediated Neuroinflammation by Modulating the TLR4/MyD88/NF-κB Pathway and Microbiome-Gut-Brain Axis in Cardiac Arrest Mice. Mol. Brain.

[17] Soeda M., Ohka S., Nishizawa D., Iseki M., Yamaguchi K., Arita H., Hanaoka K., Kato J., Ogawa S., Hiranuma A. (2023). Single-Nucleotide Polymorphisms of the PAR2 and IL-17A Genes Are Significantly Associated with Chronic Pain. Int. J. Mol. Sci..

[18] Latremoliere A., Woolf C.J. (2009). Central Sensitization: A Generator of Pain Hypersensitivity by Central Neural Plasticity. J. Pain.

[19] Ren B., Yuan Q., Cha S., Liu S., Zhang J., Guo G. (2025). Maladaptive Neuroplasticity Under Stress: Insights into Neuronal and Synaptic Changes in the Prefrontal Cortex. Mol. Neurobiol..

[20] Apkarian V.A., Hashmi J.A., Baliki M.N. (2011). Pain and the Brain: Specificity and Plasticity of the Brain in Clinical Chronic Pain. Pain.

[21] Zeng X., Sun Y., Zhiying Z., Hua L., Yuan Z. (2025). Chronic Pain-Induced Functional and Structural Alterations in the Brain: A Multi-Modal Meta-Analysis. J. Pain.

[22] Aboushaar N., Serrano N. (2024). The Mutually Reinforcing Dynamics between Pain and Stress: Mechanisms, Impacts and Management Strategies. Front. Pain Res..

[23] Fuhrmann D., Knoll L.J., Blakemore S.-J. (2015). Adolescence as a Sensitive Period of Brain Development. Trends Cogn. Sci..

[24] Kuner R., Flor H. (2017). Structural Plasticity and Reorganisation in Chronic Pain. Nat. Rev. Neurosci..

[25] Mouraux A., Iannetti G.D. (2018). The Search for Pain Biomarkers in the Human Brain. Brain.

[26] Emmert K., Breimhorst M., Bauermann T., Birklein F., Van De Ville D., Haller S. (2014). Comparison of Anterior Cingulate vs. Insular Cortex as Targets for Real-Time fMRI Regulation during Pain Stimulation. Front. Behav. Neurosci..

[27] Friebel U., Eickhoff S.B., Lotze M. (2011). Coordinate-Based Meta-Analysis of Experimentally Induced and Chronic Persistent Neuropathic Pain. Neuroimage.

[28] Geha P., Waxman S.G. (2016). Pain Perception: Multiple Matrices or One?. JAMA Neurol..

[29] Mouraux A., Diukova A., Lee M.C., Wise R.G., Iannetti G.D. (2011). A Multisensory Investigation of the Functional Significance of the “Pain Matrix.”. NeuroImage.

[30] Iannetti G.D., Hughes N.P., Lee M.C., Mouraux A. (2008). Determinants of Laser-Evoked EEG Responses: Pain Perception or Stimulus Saliency?. J. Neurophysiol..

[31] Salomons T.V., Iannetti G.D., Liang M., Wood J.N. (2016). The “Pain Matrix” in Pain-Free Individuals. JAMA Neurol..

[32] Poldrack R.A. (2011). Inferring Mental States from Neuroimaging Data: From Reverse Inference to Large-Scale Decoding. Neuron.

[33] Marek S., Tervo-Clemmens B., Calabro F.J., Montez D.F., Kay B.P., Hatoum A.S., Donohue M.R., Foran W., Miller R.L., Hendrickson T.J. (2022). Reproducible Brain-Wide Association Studies Require Thousands of Individuals. Nature.

[34] Clarke S., Rogers R., Wanigasekera V., Fardo F., Pia H., Nochi Z., Macian N., Leray V., Finnerup N.B., Pickering G. (2024). Systematic Review and Co-ordinate Based Meta-analysis to Summarize the Utilization of Functional Brain Imaging in Conjunction with Human Models of Peripheral and Central Sensitization. Eur. J. Pain.

[35] Goksan S., Hartley C., Emery F., Cockrill N., Poorun R., Moultrie F., Rogers R., Campbell J., Sanders M., Adams E. (2015). fMRI Reveals Neural Activity Overlap between Adult and Infant Pain. eLife.

[36] Fiúza-Fernandes J., Pereira-Mendes J., Esteves M., Radua J., Picó-Pérez M., Leite-Almeida H. (2025). Common Neural Correlates of Chronic Pain—A Systematic Review and Meta-Analysis of Resting-State fMRI Studies. Prog. Neuro-Psychopharmacol. Biol. Psychiatry.

[37] Cascella M., Montedoro M., Vittori A. (2026). The Medial Prefrontal Cortex as an Integrative Hub in Chronic Pain: Network Mechanisms and the Enabling Role of Artificial Intelligence. Biopsychosoc. Med..

[38] Castejón J., Chen F., Yasoda-Mohan A., Ó Sé C., Vanneste S. (2024). Chronic Pain—A Maladaptive Compensation to Unbalanced Hierarchical Predictive Processing. NeuroImage.

[39] De Ridder D., Vanneste S., Freeman W. (2014). The Bayesian Brain: Phantom Percepts Resolve Sensory Uncertainty. Neurosci. Biobehav. Rev..

[40] Büchel C., Geuter S., Sprenger C., Eippert F. (2014). Placebo Analgesia: A Predictive Coding Perspective. Neuron.

[41] Menon V. (2023). 20 Years of the Default Mode Network: A Review and Synthesis. Neuron.

[42] Heukamp N.J., Moliadze V., Mišić M., Usai K., Löffler M., Flor H., Nees F. (2025). Beyond the Chronic Pain Stage: Default Mode Network Perturbation Depends on Years Lived with Back Pain. Pain.

[43] Mussigmann T., Bardel B., Lefaucheur J.-P. (2022). Resting-State Electroencephalography (EEG) Biomarkers of Chronic Neuropathic Pain. A Systematic Review. NeuroImage.

[44] Johansson E., Xiong H.-Y., Polli A., Coppieters I., Nijs J. (2024). Towards a Real-Life Understanding of the Altered Functional Behaviour of the Default Mode and Salience Network in Chronic Pain: Are People with Chronic Pain Overthinking the Meaning of Their Pain?. J. Clin. Med..

[45] Kucyi A., Davis K.D. (2017). The Neural Code for Pain: From Single-Cell Electrophysiology to the Dynamic Pain Connectome. Neuroscientist.

[46] Yang S., Boudier-Revéret M., Choo Y.J., Chang M.C. (2020). Association between Chronic Pain and Alterations in the Mesolimbic Dopaminergic System. Brain Sci..

[47] Hiraga S., Itokazu T., Nishibe M., Yamashita T. (2022). Neuroplasticity Related to Chronic Pain and Its Modulation by Microglia. Inflamm. Regen..

[48] Ji R.-R., Nackley A., Huh Y., Terrando N., Maixner W. (2018). Neuroinflammation and Central Sensitization in Chronic and Widespread Pain. Anesthesiology.

[49] Grace P.M., Hutchinson M.R., Maier S.F., Watkins L.R. (2014). Pathological Pain and the Neuroimmune Interface. Nat. Rev. Immunol..

[50] Obara I., Tochiki K.K., Géranton S.M., Carr F.B., Lumb B.M., Liu Q., Hunt S.P. (2011). Systemic Inhibition of the Mammalian Target of Rapamycin (mTOR) Pathway Reduces Neuropathic Pain in Mice. Pain.

[51] Ma X., Du W., Wang W., Luo L., Huang M., Wang H., Lin R., Li Z., Shi H., Yuan T. (2020). Persistent Rheb-Induced mTORC1 Activation in Spinal Cord Neurons Induces Hypersensitivity in Neuropathic Pain. Cell Death Dis..

[52] Kwon M., Han J., Kim U.J., Cha M., Um S.W., Bai S.J., Hong S.-K., Lee B.H. (2017). Inhibition of Mammalian Target of Rapamycin (mTOR) Signaling in the Insular Cortex Alleviates Neuropathic Pain after Peripheral Nerve Injury. Front. Mol. Neurosci..

[53] Jones S.A., Morales A.M., Holley A.L., Wilson A.C., Nagel B.J. (2020). Default Mode Network Connectivity Is Related to Pain Frequency and Intensity in Adolescents. NeuroImage Clin..

[54] Becerra L., Sava S., Simons L.E., Drosos A.M., Sethna N., Berde C., Lebel A.A., Borsook D. (2014). Intrinsic Brain Networks Normalize with Treatment in Pediatric Complex Regional Pain Syndrome. NeuroImage Clin..

[55] Onciul R., Tataru C.-I., Dumitru A.V., Crivoi C., Serban M., Covache-Busuioc R.-A., Radoi M.P., Toader C. (2025). Artificial Intelligence and Neuroscience: Transformative Synergies in Brain Research and Clinical Applications. J. Clin. Med..

[56] Cascella M., Tracey M.C., Petrucci E., Bignami E.G. (2023). Exploring Artificial Intelligence in Anesthesia: A Primer on Ethics, and Clinical Applications. Surgeries.

[57] Woo C.-W., Chang L.J., Lindquist M.A., Wager T.D. (2017). Building Better Biomarkers: Brain Models in Translational Neuroimaging. Nat. Neurosci..

[58] Montomoli J., Bitondo M.M., Cascella M., Rezoagli E., Romeo L., Bellini V., Semeraro F., Gamberini E., Frontoni E., Agnoletti V. (2024). Algor-Ethics: Charting the Ethical Path for AI in Critical Care. J. Clin. Monit. Comput..

[59] Stitzer M.L., Schwartz R.P., Bigelow G.E. (2017). Prescription Opioids: New Perspectives and Research on Their Role in Chronic Pain Management and Addiction. Drug Alcohol Depend..

[60] Tompkins D.A., Hobelmann J.G., Compton P. (2017). Providing Chronic Pain Management in the “Fifth Vital Sign” Era: Historical and Treatment Perspectives on a Modern-Day Medical Dilemma. Drug Alcohol Depend..

[61] Barth K.S., Guille C., McCauley J., Brady K.T. (2017). Targeting Practitioners: A Review of Guidelines, Training, and Policy in Pain Management. Drug Alcohol Depend..

[62] Garland E.L., Bryan C.J., Finan P.H., Thomas E.A., Priddy S.E., Riquino M.R., Howard M.O. (2017). Pain, Hedonic Regulation, and Opioid Misuse: Modulation of Momentary Experience by Mindfulness-Oriented Recovery Enhancement in Opioid-Treated Chronic Pain Patients. Drug Alcohol Depend..

[63] Ling W. (2017). Prescription Opioid Addiction and Chronic Pain: More than a Feeling. Drug Alcohol Depend..

[64] Zis P., Daskalaki A., Bountouni I., Sykioti P., Varrassi G., Paladini A. (2017). Depression and Chronic Pain in the Elderly: Links and Management Challenges. Clin. Interv. Aging.

[65] Meier T.A., Refahi M.S., Hearne G., Restifo D.S., Munoz-Acuna R., Rosen G.L., Woloszynek S. (2024). The Role and Applications of Artificial Intelligence in the Treatment of Chronic Pain. Curr. Pain Headache Rep..

[66] Cascella M., Ponsiglione A.M., Santoriello V., Romano M., Cerrone V., Esposito D., Montedoro M., Pellecchia R., Savoia G., Lo Bianco G. (2025). Expert Consensus on Feasibility and Application of Automatic Pain Assessment in Routine Clinical Use. J. Anesth. Analg. Crit. Care.

[67] Lötsch J., Sipilä R., Dimova V., Kalso E. (2018). Machine-Learned Selection of Psychological Questionnaire Items Relevant to the Development of Persistent Pain after Breast Cancer Surgery. Br. J. Anaesth..

[68] Nickerson P., Tighe P., Shickel B., Rashidi P. (2016). Deep Neural Network Architectures for Forecasting Analgesic Response. Proceedings of the 2016 38th Annual International Conference of the IEEE Engineering in Medicine and Biology Society (EMBC).

[69] Gao X., Xin X., Li Z., Zhang W. (2021). Predicting Postoperative Pain Following Root Canal Treatment by Using Artificial Neural Network Evaluation. Sci. Rep..

[70] Gouverneur P., Li F., Shirahama K., Luebke L., Adamczyk W.M., Szikszay T.M., Luedtke K., Grzegorzek M. (2023). Explainable Artificial Intelligence (XAI) in Pain Research: Understanding the Role of Electrodermal Activity for Automated Pain Recognition. Sensors.

[71] Cascella M., Ponsiglione A.M., Santoriello V., Romano M., Amato F., Sabbatino F., Pepe S., Piazza O. (2026). Refining Multiple Artificial Intelligence Strategies for Automatic Pain Assessment Investigations (RUGGI Study): A Study Protocol. Eur. J. Anaesthesiol. Intensive Care.

[72] Home|Next2U Solutions. https://www.next2u-solutions.com.

[73] Nahin R.L., Feinberg T., Kapos F.P., Terman G.W. (2023). Estimated Rates of Incident and Persistent Chronic Pain Among US Adults, 2019–2020. JAMA Netw. Open.

[74] Prunskis J.V., Masys T., Pyles S.T., Abd-Elsayed A., Deer T.R., Beall D.P., Gheith R., Patel S., Sayed D., Moten H. (2025). The Application of Artificial Intelligence to Enhance Spinal Cord Stimulation Efficacy for Chronic Pain Management: Current Evidence and Future Directions. Curr. Pain Headache Rep..

[75] Hagedorn J.M., George T., Aiyer R., Schmidt K., Halamka J., D’Souza R.S. (2024). Artificial Intelligence and Pain Medicine: An Introduction. J. Pain Res..

[76] Najjar R. (2023). Redefining Radiology: A Review of Artificial Intelligence Integration in Medical Imaging. Diagnostics.

[77] Rockholt M.M., Kenefati G., Doan L.V., Chen Z.S., Wang J. (2023). In Search of a Composite Biomarker for Chronic Pain by Way of EEG and Machine Learning: Where Do We Currently Stand?. Front. Neurosci..

[78] Mari T., Henderson J., Maden M., Nevitt S., Duarte R., Fallon N. (2022). Systematic Review of the Effectiveness of Machine Learning Algorithms for Classifying Pain Intensity, Phenotype or Treatment Outcomes Using Electroencephalogram Data. J. Pain.

[79] Chen Z.S., Kulkarni P., Galatzer-Levy I.R., Bigio B., Nasca C., Zhang Y. (2022). Modern Views of Machine Learning for Precision Psychiatry. Patterns.

[80] Qian Y., Alhaskawi A., Dong Y., Ni J., Abdalbary S., Lu H. (2024). Transforming Medicine: Artificial Intelligence Integration in the Peripheral Nervous System. Front. Neurol..

[81] Green M., Hayley A., Gunnersen J., Nazemian V., Cabble A., Thompson S., Chakravarthy K. (2025). Transforming Chronic Pain Management: Integrating Neuromodulation with Advanced Technologies to Tackle Cognitive Dysfunction—A Narrative Review. J. Pain Res..

[82] Capetti B., Conti L., Marzorati C., Grasso R., Ferrucci R., Pravettoni G. (2024). The Application of tDCS to Treat Pain and Psychocognitive Symptoms in Cancer Patients: A Scoping Review. Neural Plast..

[83] Fernandes S.M., Peixoto J., Rodrigues P.F.S., Bártolo A. (2025). Efficacy of Neuromodulation Techniques (TMS and tDCS) in Cancer Pain Management: A Systematic Review. Int. J. Clin. Health Psychol..

[84] Piette J.D., Newman S., Krein S.L., Marinec N., Chen J., Williams D.A., Edmond S.N., Driscoll M., LaChappelle K.M., Maly M. (2022). Artificial Intelligence (AI) to Improve Chronic Pain Care: Evidence of AI Learning. Intell.-Based Med..

